# Reports of Cases Treated in the Louisville Marine Hospital

**Published:** 1840-11

**Authors:** George W. Bayless

**Affiliations:** Demonstrator of Anatomy in the Medical Institute of Louisville


### WESTERN JOURNAL

OF

### MEDICINE AND SURGERY.

---

NOVEMBER, 1840.

---

ART. I.—*Reports of Cases, treated in the Louisville Marine Hospital.* By GEORGE W. BAYLESS, M. D., Demonstrator of Anatomy in the Medical Institute of Louisville.

AMONG a considerable number of cases, treated in this house during the months of July and August, 1840, the following are considered to possess sufficient interest to merit a brief notice.

In presenting these cases, we shall not pursue the usual plan of noticing the daily condition and changes, with the prescription, for the reason that we deem it better to abbreviate by giving the general features and results, than to enter into such a detail, which is not called for by their particular character.

Case 1st. Aneurism of the Aorta.—James M'Guinnative, of Ireland, aged about twenty-six years—a stout muscular man about five feet ten inches high—a stage driver by occupation, and of intemperate habits, was admitted July 2d. Stated that he had always been healthy up to last winter—that in January last, from exposure incident to his occupation, he took a severe cold, attended with cough and pain in one side, could not recollect which—that he was puked, purged, &c. and that he got better, but not entirely well. That he was able to go about in a short time, but continued to have a dry cough, attended with some dyspnœa, &c. which prevented him from resuming his occupation. That this state of things continued through the remainder of the winter and spring, and that he began to get worse about two or three weeks before he entered the hospital.

*Symptoms when admitted.*—*Surface*—skin in good condition, inconsiderable emaciation.—*Head*, normal.—*Thorax*—cough troublesome, occasional slight dyspnœa, inconsiderable mucous expectoration, pretty good resonance, and nothing unusual discovered by auscultation. Circulation not well distributed to the extremities—pulse eighty, small and feeble—no particular examination made of the heart.—*Abdomen*—tongue slightly covered with a whitish fur; appetite not good, but the stomach retained and digested very well whatever of food was taken; bowels, in a good condition, no pain nor tenderness, upon pressure, in any part of the abdomen.

*Diagnosis* uncertain; most probably chronic bronchitis, consequent upon the cold taken last winter. Directed vegetable diet—cupping over the anterior parts of thorax—

brown mixture, and mucilaginous drink. Found him at next visit somewhat relieved of the dyspnœa, but cough still troublesome. Brown mixture continued.

There being no manifest change in two days, he was ordered ung. tart. emetic, to free pulsation on anterior thorax; brown mixture to be continued.

A copious crop of pustules was produced in a day or two, when we discovered great force in the action of the heart, communicating a considerable impulse to the hand, and attended with acute pain upon pressure. Tinct. digital. was added to the brown mixture, and cups were applied to the præcordia.

The pain was mitigated by the cupping, but this great activity in the pulsation of the heart having continued unintermittingly for several days, and the dyspnœa, dry cough, and feeble pulse, which was very small considering the activity of the heart, still continuing, led us to believe that there was contraction of the left ventriculo-aortic valves, nowithstanding we could detect no morbid alteration in the sounds of the heart. We could not only easily account for the symptoms just named, upon the supposition of this lesion of the aortic semi-lunar valves, but we were compelled to suppose this or some affection of the aorta itself, which was not then indicated by any particular symptom. Brown mixture discontiued. Pulv. digital. gr. 1—morning, noon, and night.

This was continued several days, when we were called to him early in the morning, and found him laboring under extreme dyspnœa—heart beating violently—pulse much fuller and stronger than we had ever seen it, and had only changed within a few minutes, as we were informed by the house student—surface cold, and covered with a cold perspiration; face livid and turgid with blood—eyes semi-closed, and pupils dilated—jugulars stuffed—total insensibility—brain evidently greatly engorged and oppressed with blood, and apoplexy seeming to be momentarily threatened. Before we reached the house, sinapisms had been applied to the breast and extremities, and an effort made to abstract blood: only two or three ounces could be obtained. A large orifice was immediately opened in the arm, and about thirty ounces of blood soon abstracted, with almost entire relief. His condition just described, together with the effect produced by thus rapidly diminishing the quantity of blood in circulation, confirmed us in the view which we had taken. These paroxysms continued to recur daily, and frequently two or three times a day, and always required the abstraction of blood before relief was obtained. We found that relief was afforded more speedily and with the loss of a smaller quantity of blood, which became desirable from the necessity of its frequent repetition, by opening a jugular. So satisfied was he that blood-letting was his only source of relief, that he implored it as an act of mercy, when a paroxysm was about to come on.

A few days before death, the nature of the affection became apparent from an increased fulness which was observed to make its appearance, extending two or three inches by the side of the left edge of the sternum,—commencing at the top—from a palpitation which became very obvious at the intercostal spaces of the part, and from his complaining of a "lump which choked him" about the top of the sternum. Death took place on the 8th of July, during one of the paroxysms already described.

*Examination of the Body Nine Hours after Death.*

*Head.* No cerebral symptoms having been observed since opening the jugular had been resorted to, this cavity was not examined.

*Thorax—Lungs.*—Pulmonary veins partially engorged with very black blood—normal in every other respect, save interlobular emphysema which was pretty generally present throughout the lungs. *Heart.*—Right auricle and ventricle contained fibrinous concretions—the auricle containing also a coagulum of black blood—while the ventricle was almost entirely filled with an opaque, firm concretion, which adhered with some firmness to the columnæ carneæ; there was also a dark coagulum in the left auricle, but the left ventricle was empty. No particular lesion of the heart was discoverable, save a slight hypertrophy, without dilatation, of the left ventricle. *Aorta.*—At its arch we found an aueurismal sac, a cylindroid in shape, about two and a half inches in length, and two in diameter. It contained a small opaque coagulum, and probably others, but wishing to preserve it entire we did not lay it open. By the whole of its posterior surface it adhered very firmly, indeed inseparably, to the anterior surface of the trachea, just above its bifurcation. The coats of the sac were entire.

*Abdomen.*—No lesion, of consequence, of any its viscera.

The heart and aorta with the adherent trachea were placed in the Pathological Cabinet of the Louisville Medical Institute, where they may be seen.

Case 2d. Dysentery.—M'Donald, native of Ireland, aged twenty-seven years, (a mariner,) was transferred from the surgical to this ward on the 12th of July. He had been about two months in the surgical ward, under treatment for syphilis; and two days before he was transferred he was severely attacked with dysentery—having from ten to twenty evacuations in the twenty-four hours, of blood and mucus, producing great prostration, and attended with considerable tenesmus, but unaccompanied by any tormina, or febrile action. These symptoms continued at the time of his transfer. We will remark that the disease was prevailing to some extent in the city at the time, but this was the only case that made its appearance in the hospital.

℞. Calom. gr. ss., opii gr. ¼, ipecac. gr. i. ft. pilul., one every three hours. ℞. Tinct. opii gtt. xxv., mucilag. Amyli. f℥ iii. ft. enema, to be repeated pro re nata. Cupping over both iliac regions; mucilaginous drink and farinaceous diet.

Under this treatment, with the addition of blisters to the iliac regions continued for several days, the disease seemed to subside rapidly. The number of evacuations was reduced to three or four a day; they changed to an opaque whitish mucus, the tenesmus entirely subsided, and slight ptyalism was induced. Directed—Oleag. mixt. f℥ ss., every two hours until the bowels should be gently opened. Anodyne enema, diet and drinks continued.

Copious fecal evacuations, mixed with a very small quantity of the opaque mucus, were produced by the oleaginous mixture. Strength pretty good; some appetite. ℞. opii gr. ¼, ipecac. gr. i. ft. pil. one every four hours. Diet and drink continued.

There was decided and progressive improvement for several days in the character of the evacuations, in strength and appetite; but still some diarrhœa. Dover's powder grs. v. every six hours. Chicken broth—mucilag. drink. Continued to improve for some days, but the diarrhœa still obstinately continued, and the evacuations became of a brown watery character, mixed with some fecal matter, and very offensive. ℞. acetat. plumbi grs. ii., opii gr. ¼, ft. pil., one every three hours. Anodyne enema—farinaceous diet—mucilaginous drink.

From this time the diarrhœa continued; he gradually sunk. Hope's camphor mixture was employed, but he complained of its burning him; ammoniated julep, wine whey, sinapisms, &c., but he continued to sink, and died exhausted on the 27th of July.

*Examination of the Body Fifteen Hours after Death.*

*Exterior.*—Extreme emaciation. A bubo in each groin—an ulcer about the middle of the hypogastric region, leading to a cavity between the oblique and rectus, and transversalis abdominis muscles somewhat circular in its shape and three or four inches in diameter, having a branch extending down over the left upper branch of the pubis, so as to penetrate three or four inches between the adductor muscles. It contained a brownish offensive matter; and that portion of the pubis over which it passed was carious.

*Head*—Not examined.

*Thorax.*—Viscera normal.

*Abdomen.*—No trace of peritoneal inflammation could be discovered. *Stomach.*—Mucous membrane of its cardiac extremity thickened, and in a state of deep hyperæmia. *Small Intestines* normal. *Large Intestine.*—A number of ulcers, throughout its whole extent, mostly circular in form, and varying in size from two or three lines, to eight or nine in diameter. They presented a dark blackish appearance, the smaller penetrating only through the mucous coat, and the larger, having slightly elevated edges, penetrating through the subjacent tissue, leaving only the peritoneal entire. The mucous membrane was of a pale yellow color, softened throughout and deficient in many places. The deficiences increased from the cœcum to the rectum, there being only some fragments of the membrane left at the sigmoid flexure, and entirely wanting in the rectum. At the place where it had lost the subjacent cellular tissue it presented a purple or blackish hue. Liver, spleen, and kidneys normal.

The large intestine and carious pubis were preserved for the Pathological Cabinet of the Medical Institute.

Case 3d. Dropsy.—M'Kay, a native of Kentucky, aged twenty-seven years—a marine—temperate habits, was admitted July 20th. Stated, that he had an attack of yellow fever in Natchez, in the fall of '39; that during convalescence from that attack he was seized with intermittent fever, which continued through the winter; that during the spring it several times ceased, and returned again at longer or shorter intervals, and that three weeks before entering the house his abdomen and extremities commenced swelling.

*Condition when admitted.*—Very weak, unable to sit up. *Surface*, extremely pale; skin distended by general anasarca, extending from head to feet; face greatly swollen; parietes of thorax and abdomen also implicated; lower extremities prodigiously large, upper not so large. *Head*, normal. *Thorax*, respiration performed easily; could discover no effusion into that cavity. *Heart*, normal; pulse about eigthy, and of tolerable strength. *Abdomen* immensely swollen by serous effusion, as evinced by a very obvious fluctuation. Digestion feeble; bowels constipated; urine in moderate quantity; could not discover the condition of the liver or spleen, by physical exploration, so great was the abdominal enlargement. Elaterium gr. ss., every two hours, until the bowels should be freely purged—farinaceous diet.

This treatment was continued a week, tolerably free fecal evacuations having been produced, and a slight diminution in the size of the lower extremities.

Failing about this time to make our usual daily visit, we found on the second day, so great a diminution in the size of the face and abdomen that we were unable to recognize our patient until informed by himself of the nature of his disease. In the interval he had very copious watery evacuations, mixed with fecal matter, amounting to near a gallon in twenty-four hours, but yet his strength was not lessened. Some irritation of the stomach. Suspend the use of the medicine one day.

The elaterium was commenced on the second day and was continued for five or six days; at the end of which time, or about two weeks from his admission, the abdominal effusion seemed entirely gone, the swelling of the face and general surface had disappeared, there remained only a vestige of the swelling of the lower extremities, and his strength was so improved that he was able to walk about the ward. Discontinue medicine—bandage to lower extremities—nutritious diet.

From this time forward he went on to improve, without interruption, save one paroxysm of intermittent, and when the ward was transferred to Dr. Caldwell, on the 1st of September, he was on the use of the precipitated carbonate of iron, and was able to go about the yard, and perform the duties of assistant nurse.

Case 4th. Dropsy.—On the 17th of August, was admitted Hartshorne, native of New York, aged forty-seven years, a laborer—temperate habits. Stated, that he had for several years been subject to attacks of rheumatism; that about six weeks before his admission he was seized with acute rheumatism, affecting more particularly the knees; that he employed no physician, and used no remedial means, save perhaps some local stimulants; that the "pain in his knees" subsided in about a month; that shortly after he was seized with a very acute pain in the region of the heart, which continued three days, and spontaneously subsided; that about the same time or immediately subsequent to it, his feet and legs, and abdomen commenced swelling, and continued to do so until the the time of his admission into the hospital.

*Condition when admitted.*—*Head*, normal. *Thorax*—respiration natural. Resonant throughout, save great dullness upon percussion, over a space covering the præcordial region proper, extending from the second to the seventh intercostal space, and about six inches in width. *Heart*, was found beating with considerable force, the impulsion being felt at the third intercostal space, above the nipple, instead of, as usual, at the cartilage of the fifth rib; no morbid sound could be detected by auscultation; pulse about ninety and very feeble. *Abdomen*, greatly distended by serous effusion; fluctuations very plain, appetite not good, digestion feeble, bowels loose, having four or five watery evacuations in twenty-four hours; urine in natural quantity; could not ascertain the condition of the liver and spleen. Lower extremities œdematous.

*Diagnosis.*—Contraction of the ventriculo-aortic semi-lunar valves, probably by the deposit of lymph at the time of the acute pain complained of, when metastasis took place. Probably hydropericardium.

*Ascites* and œdema of the lower extremities, dependant upon the obstructed circulation. From the diarrhœa we thought that absorption of the effused fluid had commenced, and was being thrown off by the bowels. Elaterium gr. ss., every two hours—farinaceous diet.

The treatment was continued four or five days, with the effect of producing almost a constant thin, watery discharge from the bowels, with an obvious diminution in the size of the abdomen and lower extremities, and without much loss of strength. In consequence of some irritation of the stomach and bowels, the use of the medicine was suspended thirty-six hours. It was then re-commenced and continued until the 26th, with the same effects; when the ascites and œdema were almost entirely gone, and the dull præcordial space was not so large. The use of the medicine was again suspended, but the discharges from the bowels continued, and when he was transferred with the other patients on the 1st of September, there was scarcely a relic of the ascites and œdema; strength improving; free from pain, and in a fair way for at least a temporary recovery. From the active pulsation of the heart and the feeble pulse still continuing, there probably exists the structural lesion of the aortic valves spoken of, which will render him liable to dropsy again.

Case 5th. Neuralgia relieved by the extraction of a Tooth.—In the American Journal of Medical Sciences, for August, 1840, p. 509, Mr. G. B. Fundenberg, Dentist at Pittsburg, makes some observations on facial neuralgia, in which he asserts with most apparent confidence, that at least two thirds of the cases of this affection "are caused by pressure or other local irritation of the nerve." In support of his positon he gives three cases. In the first—a case of fourteen years standing, the anterior maxillary foramen, the point at which the patient had complained of most pain, was nearly closed; most probably by an osseous deposit, consequent upon the formation of an alveolar abscess contiguous to it. The foramen was exposed by an incision and drilled out, by which the nerve was freed from pressure, and the patient entirely relieved from his painful malady. In the second, a well marked case, the suspicion arose that it proceeded from some affection of the teeth; and upon tapping a superior bicuspis the patient screamed out with pain. An immediate cure was effected by extracting the tooth, on the end of the fang of which was found " a large exostosis." In the third—a case of two years standing, in which the patient had complained of " a singular pain in the side of the head, accompanied by a darting pain in the lower jaw," and which gradually extended down the neck and side, a similar effect as in the second case was produced by tapping an inferior cuspidatus—the tooth was extracted—" a large osseous deposit was found on the fang"—and the patient was relieved.

We are by no means prepared to assent to this opinion of Mr. F.—that two thirds of the cases of facial neuralgia proceed from the causes above mentioned ; nor are we disposed to controvert his position ; for it is very difficult, in a disease the cause of which has been admitted by all writers on the subject to be so obscure, to fix any proportion of the kind. It will certainly require much additional evidence to establish the truth of his position. Doubtless however, he is correct in as far as he supposes some cases to be attributable to such cause ; aud we can give a case which we consider in point.

In the early part of July last, before the number of the Journal containing Mr. F.'s article was published, we were called to see Sidney, a negro girl, belonging to a gentleman of this city, who had been suffering a day or two with a severe "pain in her face." She is about 20 years old, stout, and has been always healthy, save that recently she has suffered with some slight catamenial derangement. At the time we were called to see her, there was no derangement of her general health, and she complained only of pain in the left side of her face, which was most severe about the articulation of the jaw, and extended anteriorly to the angle of the mouth, and nearly to the nose—above, a short distance upon the side of the head, and below, upon the neck as far back as the edge of the trapezius muscle, and nearly down to the point of the shoulder. It was paroxysmal in its character—was much the most severe at night—and unattended by any febrile excitement, tumefaction, heat, &c., showing it to be clearly a case of neuralgia, and not of rheumatism. A purgative had been given before we saw her, and we directed strong counter-irritation by means of a small blister by the concentrated spts. ammon. over the root of the facial nerve; and 10 drops of the acet. tinct. of opium, to be repeated as might be necessary.

This treatment was continued for several days, and the severity of the paroxysms was somewhat mitigated by the repetition of the circumscribed blister, which was generally drawn in two or three minutes; but no material improvement was effected, and she was directed to rub the face frequently and freely with veratria ointment, containing fifteen grains to two drachms of lard. Several days elapsed with no relief, save the temporary mitigation of pain by the blister; and a pill containing the twelfth of a grain of veratria was directed to be taken every four hours, together with a continuation of the ointment, &c. This treatment was kept up until the twelfth or fourteenth day of the attack, without any additional amendment, when upon examining her mouth we found several decayed teeth in both the upper and lower jaw of the affected side. Thinking that possibly the condition of the teeth might have caused some irritation of the second or third branch of the fifth pair of nerves, which could be transmitted to the portio dura by the free and anastamosis existing between them, and thus have produced the neuralgia, we sent her to a dentist, who found that some pain was produced in several of the decayed teeth by tapping on them, and severe pain in the first molar of the upper jaw by the same means. He extracted the tooth, and found at the extremity of its root a sac about the size of a pea, which had been filled with pus, but was ruptured by the operation. The root itself was carious; so also were several fangs that were extracted. Some soreness remained for several days, but about six weeks have elapsed, and she has had no return of the neuralgia.

*September* 10*th*, 1840.

